# Supramolecular Structure of Sulfonamide-Substituted Silatranes: Quantum Chemical DFT Calculations

**DOI:** 10.3390/ijms252211920

**Published:** 2024-11-06

**Authors:** Nina N. Chipanina, Sergey N. Adamovich, Arailym M. Nalibayeva, Yerlan N. Abdikalykov, Larisa P. Oznobikhina, Elizaveta N. Oborina, Igor B. Rozentsveig

**Affiliations:** 1 A.E. Favorsky Irkutsk Institute of Chemistry, Siberian Branch of the Russian Academy of Sciences, 1 Favorsky Street, 664033 Irkutsk, Russia; nina_chipanina@irioch.irk.ru (N.N.C.); l_oznobikhina@irioch.irk.ru (L.P.O.); oborina@irioch.irk.ru (E.N.O.); i_roz@irioch.irk.ru (I.B.R.); 2 D.V. Sokolsky Institute of Fuel, Catalysis and Electrochemistry, 142 Kunayev Street, Almaty 050010, Kazakhstan; a.nalibayeva@ifce.kz (A.M.N.); y.abdikalykov@ifce.kz (Y.N.A.)

**Keywords:** silatrane, sulfonamide, X-ray, FTIR, DFT calculation

## Abstract

The supramolecular structure of the crystal products–*N*-[2-chloro-2-(silatranyl)ethyl]-4-nitro-benzenesulfonamide **4d** and *N*-chloro-*N*-[2-chloro-1-(silatran-1-yl-methyl)ethyl]benzene-sulfonamide **5a** was established by X-ray diffraction analysis data, FTIR spectroscopy and DFT quantum chemical calculations. Their crystal lattice is formed by cyclic dimers with intermolecular hydrogen NH∙∙∙O-Si bonds and CH∙∙∙O=S short contacts. The distribution of electron density in the monomers was determined using quantum chemical calculations of their molecular electrostatic potential (MESP) in an isolated state (in gas) and in a polar medium. The transition from covalent N–Si bonds in crystal compounds and polar medium to non-covalent N∙∙∙Si bonds happened while performing the calculations on the monomer molecules and their dimers in gas. The effect of intermolecular interactions on the strength of the N–Si and N∙∙∙Si bonds in molecules was evaluated through calculations of their complexes with H_2_O and DMSO.

## 1. Introduction

Chelate silicon compounds are widely known. So, numerous reviews and monographs are devoted to their synthesis and stereoelectronic structure [1,2,3,4,5]. The dative bond D→Si (D = O or N) is responsible for unique structural and spectral features of these compounds and predetermines peculiarities of their stereodynamic behavior and reactivity. Such silicon derivatives find application as synthons and catalysts in organic and medicinal chemistry, as well as in materials science [1,2,3,4,5,6].

**Figure 1:**
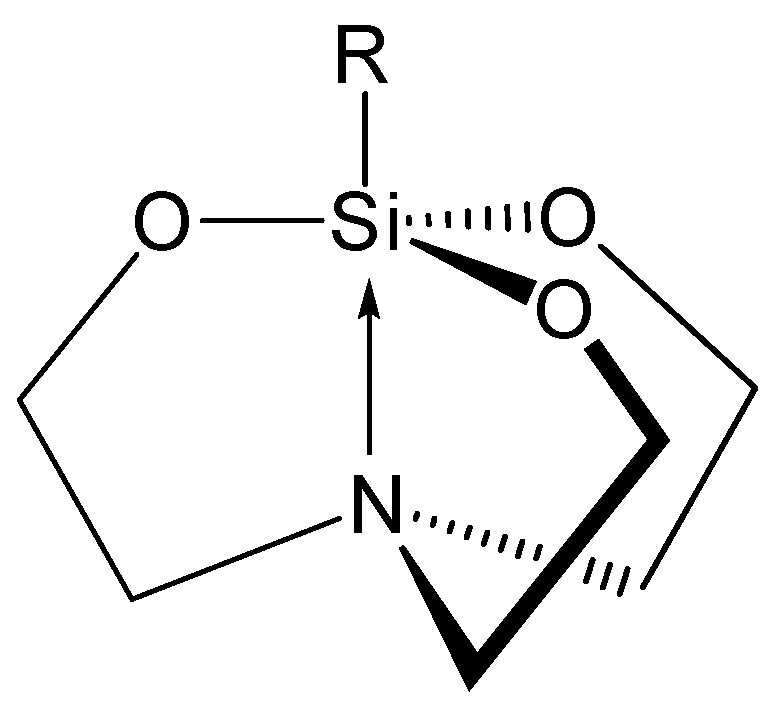
Chemical structure of silatranes.

A special class of these compounds is presented by silatranes, R-Si(OCH_2_CH_2_)_3_N. The latter contain an intramolecular transannular bond N→Si, which imparts them a tricyclic structure (Figure 1) [1,2].

The especial structure of silatranes is a cause of their unusual physical and chemical properties. For example, the strong electron-donating effect of the silatranyl group ensures high reactivity across the substituent (R) [6,7]. In addition, it has been shown that the reactivity of silatranes depends on their basicity. For instance, nucleophilic centers in silatranes have been found to be oxygen atoms (O). They are capable of forming hydrogen bonds with solvents (CHCl_3_) and donor–acceptor complexes with Lewis acids (ZnCl_2_, AlBr_3_ and TiCl_4_). It should be noted that the basicity of the nitrogen atom is very low [8].

**Figure 2:**
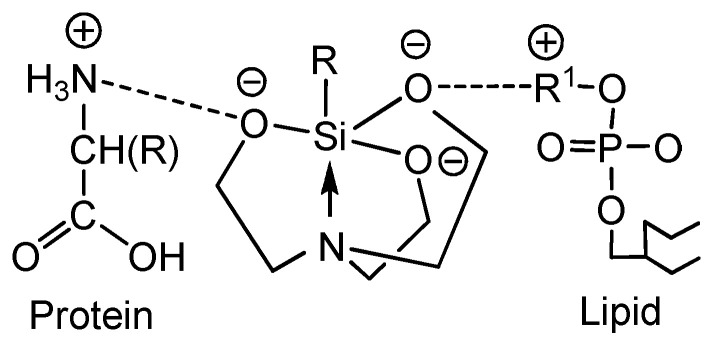
Adsorption of silatranes on cell membranes.

Finally, many silatranes are known to exhibit high and diverse biological activity, e.g., antimicrobial, antiparasitic, antihepatitis B, antitumor, immunomodulatory, growth stimulating, etc. [9,10,11,12,13,14]. The strong physiological action of silatranes can be explained by their easy adsorption on cell membranes due to hydrogen bonds and dipole–dipole interactions with polar groups of proteins and lipids (Figure 2) [10,12]. Afterwards, the silatranes penetrate into a living cell.

The physiological activity of silatranes is defined not only by the silatranyl architecture, but also depends strongly on the R substituent at the silicon atom [6,7,8,9,10,12,13,14]. R-functionalization (hybridization) of the silatranes using directed synthesis, molecular modeling, molecular docking and DFT studies to obtain the most effective bioactive compounds including drugs is widely employed [9,10,11,12,13,14]. Comparison of known medicines with hybrid analogs, e.g., sulfonamide hybrids [15], indicates that the latter are more efficient [16].

**Scheme 1:**
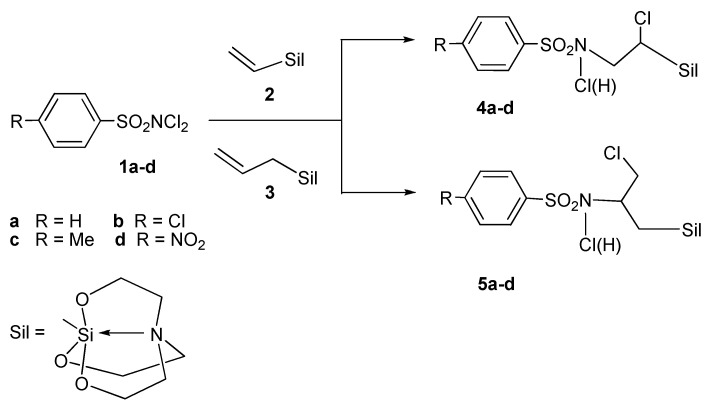
Synthesis of silatranes **4a-d** and **5a-d**.

Recently, a series of new sulfonamide-bridged silatranes, (**4a-d**) and (**5a-d**), has been synthesized by the reaction of *N*,*N*-dichloroarylsulfonamides (**1a-d**) with 1-vinylsilatrane (**2**) and 1-allylsilatrane (**3**) [17]. The compounds were found to exhibit antimicrobial activity against pathogenic bacteria that can cause sepsis, listeriosis, pneumonic and bubonic plague (Scheme 1).

Products **4** and **5** (with N-Cl bond) are poorly soluble and unstable. Therefore, they were reduced by treatment with Na_2_SO_3_ to obtain stable compounds with N-H bonds (Scheme 1). Single crystals of *N*-[2-chloro-2-(silatranyl)ethyl]-4-nitro-benzenesulfonamide **4d** (with N-H bond) and *N*-chloro-*N*-[2-chloro-1-(silatran-1-yl-methyl)ethyl]benzenesulfonamide **5a** (with N-Cl bond) were grown from CHCl_3_ solution. The structure of these silatranes was determined by X-ray diffraction analysis [17].

According to X-ray diffraction data, the length of the dative bond in chelate silicon compounds is commonly close to the sum of the covalent radii of its atoms. This, together with the results of the quantum theory of atoms in molecule (QTAIM) analysis, indicates the covalent nature of these bonds. There are both covalent (CB) and non-covalent (NCB) bonds [18,19,20,21]. However, some issues concerning the effect of structure and environment on the formation of N→Si and O→Si chelate compounds still remain open. At the same time, intramolecular non-covalent bonding is essential for different areas ranging from molecular biology to energetic materials. In addition, it was reported on receptors containing such a bond [18,22].

In 2013, a new concept in investigations of non-covalent interactions of 14 group elements (Si, Ge, Sn and Pb) with neutral or negatively charged Lewis bases was proposed [23,24]. According to this concept, such interactions are described by “σ-hole” [25], which represents a positively charged region localized on the back side of the 14 group atoms along the extension of the relevant covalent bond. The concept operates with a new term (“tetrel bond”), i.e., a bond between the “σ-hole” of the 14 group elements with the lone electron pairs (LEPs) of nitrogen or oxygen atoms. The non-covalent interactions have been categorized based on the interacting atoms containing tetrel bonds [23,26,27,28,29,30,31,32,33,34,35,36] and hydrogen bonds [30,31]. DFT quantum calculations have allowed the ability of nitrogen-containing bases to form non-covalent tetrahedral bonds to be determined [32] and the factors influencing their strength in pnictogens to be established.

X-ray structures of a number of silatranes with N–Si bonds of various lengths were analyzed by comparison of the obtained geometric parameters with the results of quantum chemical calculations of these compounds in a crystalline state and in gas [33]. The effects of substituents at the silicon atom and in the silatranyl core on the length of the N–Si bond were evaluated. The N–Si distances in 19 studied compounds (1.96–2.17 Å) are close to the sum of the covalent radii of the nitrogen and silicon atoms (1.82 Å [34]) and are tetral covalent bonds (TCBs). Only in the compound with a methyl substituent at the silicon atom and three CHMe at the nitrogen atom, the bond length (2.325 Å) exceeds this sum by 0.50 Å and the bond is tetral non-covalent (TNCB). The presence of strong electronegative substituents at the silicon atom significantly shortens the length of the dative contact, while the substituents in the silatranyl backbone, on the contrary, lengthen this distance. The replacement of ethylene fragments in “ordinary” silatranes X-Si(OCH_2_CH_2_)_3_N (X = H, Me, F) with phenylene fragments (i.e., the transition to tribenzilatranes) noticeably elongates the N∙∙∙Si (*d*_SiN_) contact (*d*_SiN_ = 2.19 Å for Ph-Si(OCH_2_CH_2_)_3_N and *d*_SiN_ = 2.33 Å for Ph-Si(OC_6_H_4_)_3_N) [35]. In both cases, such elongation of the dative bonds allows for considering these tetel bonds as non-covalent ones. The transition of intramolecular covalent N–Si bonds to non-covalent ones also occurs in crystalline silanes during intermolecular interactions [36].

The aim of this work was to establish the supramolecular structure of crystalline sulfonamide derivatives of silatranes **4d** and **5a** by comparing the data of X-ray diffraction analysis with the results of quantum chemical calculations of dimeric fragments in gas and polar medium. DFT quantum chemical calculations of monomeric molecules and their dimers were performed to evaluate the mutual effects of the geometry and electronic structure of these compounds in order to gain insight into the complex interactions between the synthesized silatranes, targets and diseases. The effects of substituents on the N–Si distance were assessed in monomers **4d**, **5a** and model **6** and **7** with substitutes CF_3_SO_2_ and MeSO_2_, respectively. The electron distribution on the molecular surface was determined by calculating the molecular electrostatic potential (MESP), which indicates possible directions of nucleophilic and electrophilic attacks, and hence, possible routes of interactions with proteins of organisms. Given that the antimicrobial activity of silatranes was studied in a 1% aqueous solution of DMSO under biomimetic conditions (H_2_O, 25 °C), the calculations concerning the complexes were performed in gas and polar medium of their complexes with H_2_O and DMSO.

## 2. Results and Discussion

### 2.1. X-Ray Diffraction Data and FTIR Analysis

**Figure 3:**
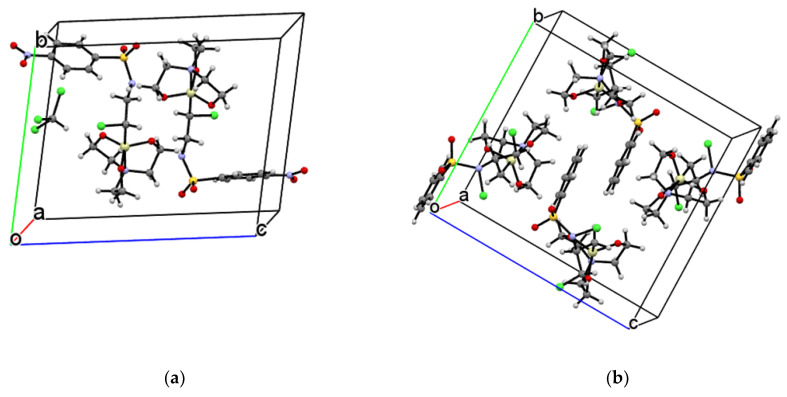
Independent cell of compounds **4d** (**a**) and **5a** (**b**).

According to X-ray diffraction analysis, the independent crystal cell of silatrane **4d** contains two of its own molecules and a molecule of chloroform (Figure 3a).

**Figure 4:**
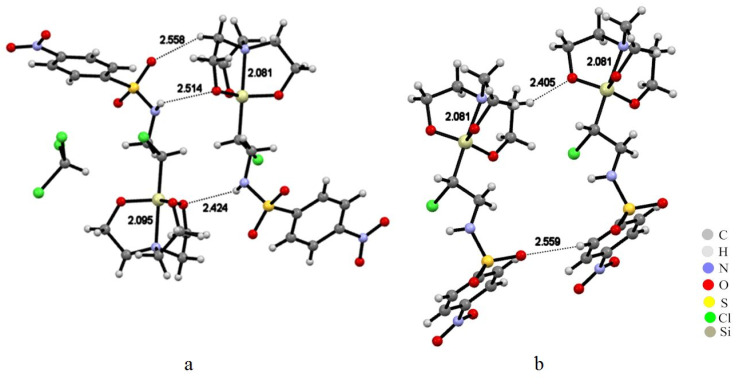
Molecular structure of compound **4d** dimers: **4d**-di1 (**a**) and **4d**-di2 (**b**). Length of bonds is given in angstroms (Å).

The silatrane molecules form a cyclic dimer **4d**-di1 involving two intermolecular hydrogen bonds (HB) NH∙∙∙O-Si (2.424 and 2.514 Å) and short contact CH∙∙∙O=S of 2.558 Å (Figure 4a).

**Figure 5:**
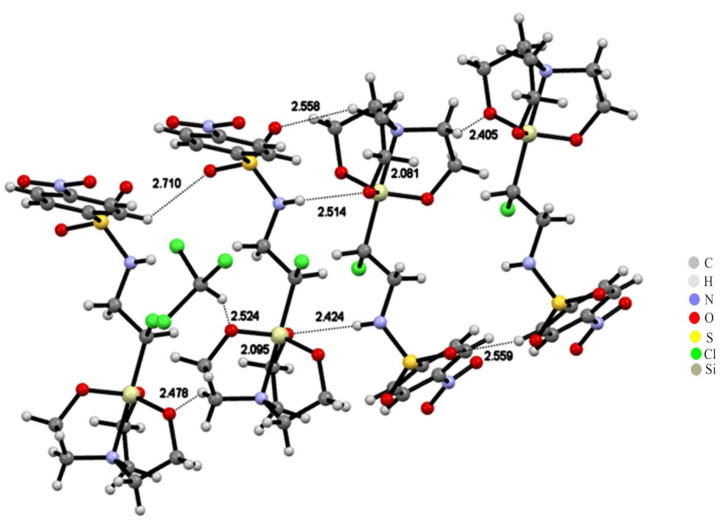
A fragment of four molecules of the crystal structure of compound **4d**. The length of bonds is given in angstroms (Å).

The length of these HBs is slightly less than the sum of the van der Waals radii of the hydrogen and oxygen atoms (2.72 Å [37]). The length of the dative N–Si bonds (2.095 Å) in the silatranyl cycle, the oxygen atom of which participates in the formation of a weak intermolecular bond (CH∙∙∙O–Si 2.524 Å) with the chloroform molecule (participant of the crystallization process), is 0.014 Å longer, and hence, weaker, than in the cycle of the second molecule (2.081 Å) that does not form the bond with chloroform. The difference in the lengths of two identical hydrogen bonds of the dimer, which manifest themselves in elongation upon the interaction of one of its components with chloroform, is obviously due to the effect of chloroform. A fragment of the crystal structure of compound **4d**, consisting of four monomeric molecules (Figure 5), evidences the formation of dimers **4d**-di1 and **4d**-di2 (Figure 4) owing to the short contacts CH∙∙∙O–Si 2.405, 2.478 Å and CH_Ph_∙∙∙O=S 2.559, 2.710 Å with neighboring molecules in both directions along the c axis.

**Figure 6:**
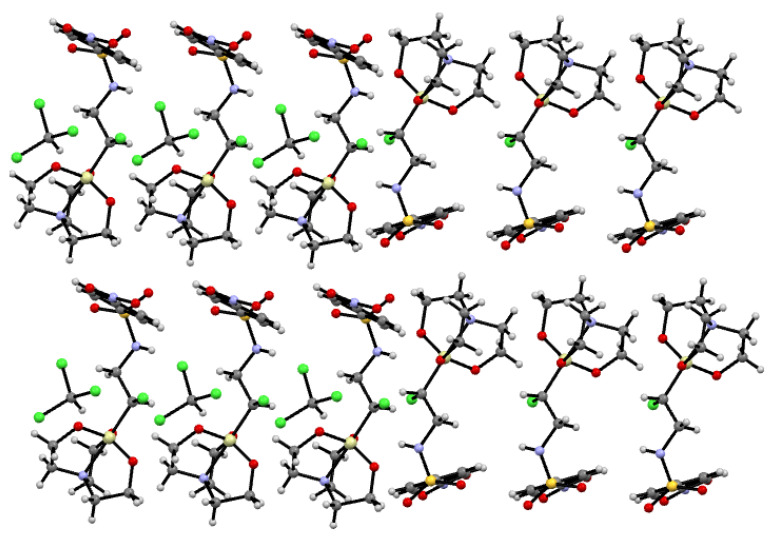
A fragment of the crystal lattice of compound **4d**.

These contacts are longer in the c axis direction, where the dimers incorporate a chloroform molecule. Consequently, infinite chains consisting of **4d** molecules are formed, in both directions from the independent cell along the c axis. These chains differ in their orientation, the participation of the chloroform molecule, and the length of not only the same type of short contacts, but also the N–Si bonds in accordance with the structure of central **4d**-di1 dimer. The chains represent layers located along the b axis, hydrogen bonds or short contacts between them being absent (Figure 6).

The FTIR spectrum of crystal compound **4d** was taken on the KBr pallets in film obtained by evaporation from the solution in CHCl_3_. The bands were assigned in accordance with the values of the vibrational frequencies calculated in the polar medium, used without a scaling factor. In the IR spectrum, the bands of the stretching vibrations of the N–Si bond were observed at 590–570 cm^−1^, while the intense band at 1090 cm^−1^ is attributable to the ν(O–Si) vibrations [6,38]. The calculated frequencies at 587, 571 and 1142, 1126 cm^−1^ belong to these vibrations. The bands at 1529 and 1350 cm^−1^ are assigned to ν_as_(NO_2_) and ν_s_(NO_2_) vibrations, respectively. A band of the stretching vibrations of associated NH groups was detected at 3250 cm^−1^. Its difference with the vibration frequency of free NH groups (ν(NH) 3370–3360 cm^−1^) in the spectra of sulfonamide derivatives solution in methylene chloride [39] was 110–120 cm^−1^. However, this difference is much less than the calculated value Δν(NH) (190 cm^−1^) between the values ν(NH) 3522 and 3332 cm^−1^ related to monomer **4d** and dimer **4d**-di1, respectively. This is due to the large difference between the length of the NH∙∙∙O-Si intermolecular bonds (2.424 and 2.514 Å) of the **4d**-di1 dimer in the crystal lattice (therefore close to the value in the film), and that calculated in a polar medium without a chloroform molecule (1.991 Å).

**Figure 7:**
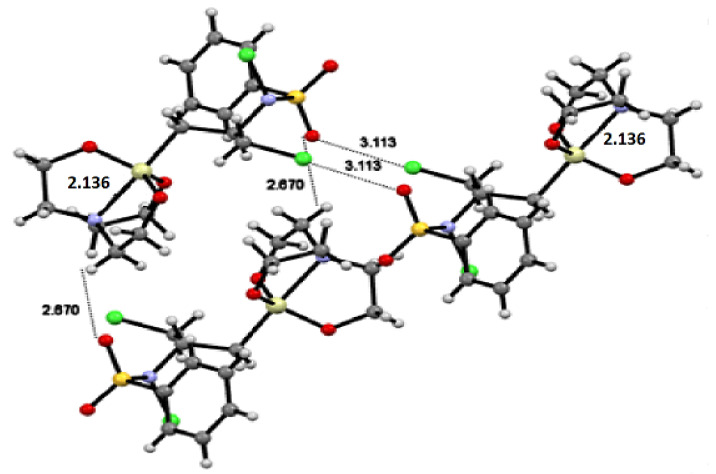
Molecular structure of dimers **5a**: **5a**-di1, **5a**-di2 in fragment of crystal lattice. Length of bonds is given in angstroms (Å).

The crystal cell of compound **5a** contains four independent molecules (Figure 3b). At the same time, its crystal lattice includes cyclic dimers **5a**-di1, formed via two short contacts CH∙∙∙O=S 2.670 Å. With these dimers, short contacts C-Cl∙∙∙O=S 3.113 Å afford cyclic dimers **5a**-di2 (Figure 7). In all molecules of the crystal, the intramolecular bonds N–Si 2.136 Å are 0.04 and 0.06 Å longer than those in the crystal compound **4d**, but they are also covalent tetrels, though less strong.

### 2.2. Electron Density and MESP

**Figure 8:**
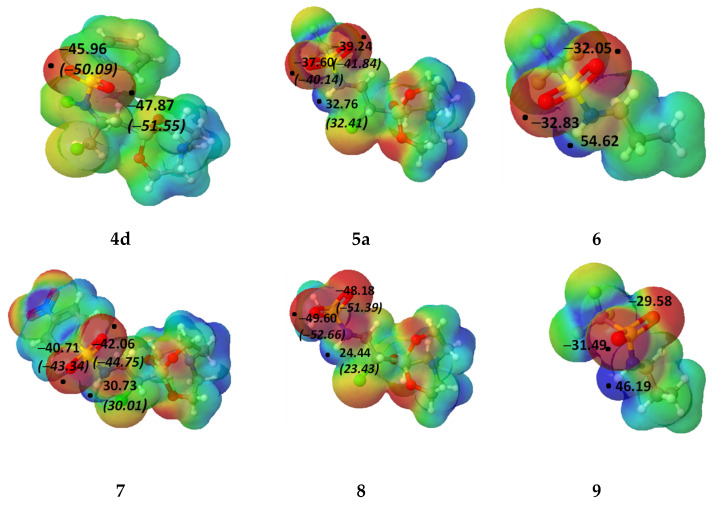
*V*_min_ and *V*_max_ (kcal/mol) CDD maps of compounds **4d**, **5a**, **6**–**9**, calculated in gas phase and DMSO (in brackets): charge accumulation—red, charge depletion—blue, yellow, green between red and blue.

TB formed by the IV group elements with the nitrogen LP allows for estimating the transformation of a covalent TB to a non-covalent one depending on the substituent and the effect of weak intermolecular interactions and the external electric field [40]. The electron density distribution in compounds **4d**, **5a**, **6** and **7** is shown in Table 1 and on the MESP maps (Figure 8), and is a good indicator of which site or region in the molecule the approaching electrophile or nucleophile is initially attracted to [41].

From gas phase calculations of silatrans having NTB N∙∙∙Si, the most negative MESP points (Vmin), from −50 to −40 and from −35 to −21 kcal/mol, are located, respectively, at the oxygen atom of O=S and O-Si groups. In addition, for compound **4d**, the points of −33 kcal/mol are found at the oxygen atoms of the nitro groups. The most positive values *V*_max_ of 33–30 kcal/mol are observed near the methylene and NH group protons. Further polarization at the PCM calculation in DMSO solution strongly shortens the N∙∙∙Si distance, up to the formation of covalent O–Si TBs. This is accompanied by an insignificant (~1 kcal/mol) decrease in the positive MESP of the NH group proton and increase (by 5–8 kcal/mol) in the MESP of the CH_2_ group protons. The negative MESP for the oxygen atoms of the O=S and O-Si groups augments in the same range. A very small positive MESP (2–7 kcal/mol) and a slightly larger negative MESP (from −6 to −16 kcal/mol) are located in the region of the silicon and chlorine atoms of the CH_2_Cl group. It should be noted that for some oxyalkyl derivatives of trifluoromethanesulfonamide, the positive MESPs of the NH group proton, calculated in the gas, range 52–68 kcal/mol and change depending on the substituent and the conformation of the molecules [42]. The MESPs were calculated for two model compounds that do not contain a silatranyl fragment, CF_3_SO_2_NHCH_2_CHClCH_3_ (**8**) and CF_3_SO_2_NHCH_2_CH_2_CH_3_ (**9**). The positive MESP of the NH group proton of compound **8** increases to 46.19 kcal/mol, and that of compound **9,** which does not contain a silatranyl fragment and chlorine atom, increases to 54.62 kcal/mol. The negative MESP at the O=S group oxygen remains at a level of 30–31 kcal/mol. Thus, it can be concluded that both the silatranyl cycle and the chlorine atom exert an electron-donating effect on the NH group that reduces the values of the positive MESPs.

### 2.3. Intermolecular H-Bonds and Intramolecular Covalent Tetrel and Non-Covalent Tetrel Bonds at the ***4d*** and ***5a*** Complexes with H_2_O and DMSO Molecules

**Figure 9:**
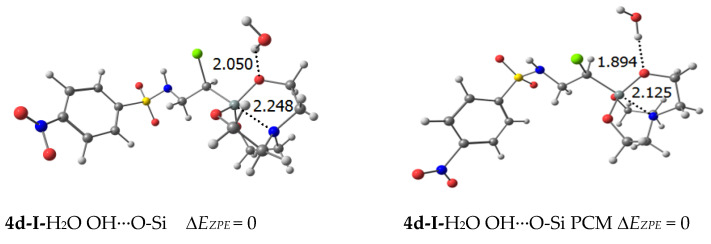

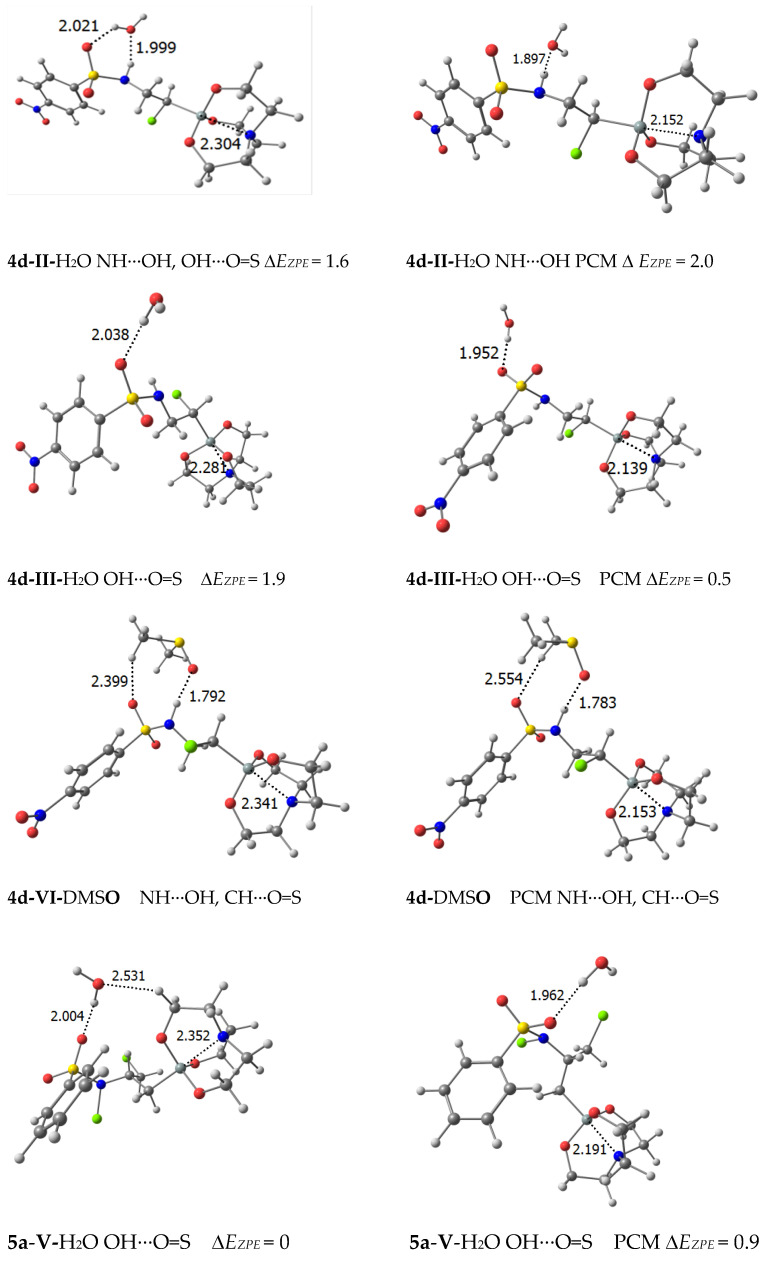

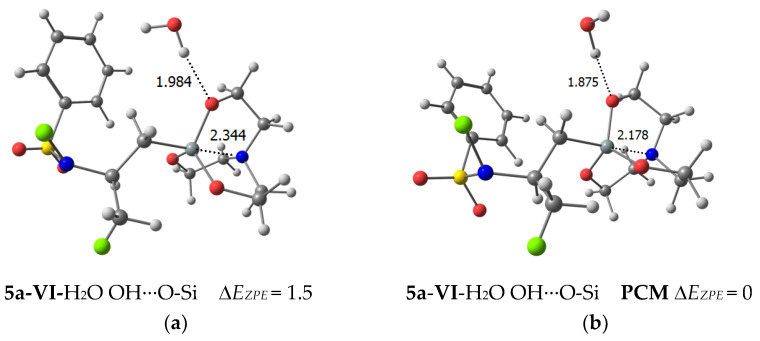
Calculated in gas and polar medium (PCM) of monomers **4d** (**a**), **5a** (**b**) and their complexes with H_2_O molecule. Length of bonds is given in angstroms (Å), Δ*E_ZPE_* in kcal/mol.

The effect of a water molecule as a donor and/or acceptor of electrons, which is added to different centers with the highest positive or negative MEP value, on the intramolecular N–Si bond of monomers **4d** and **5a** is evaluated by comparing the lengths of these bonds in the initial molecules and their water complexes using the calculations in a gas and in a polar medium (Table 1, Figure 9). The calculations (in gas) of the interaction between water molecules and compound **4d** (Figure 9a) show that the most energetically stable complex is formed by the OH∙∙∙O–Si H-bond with a length of 2.050 Å. In this case, the N∙∙∙Si bond becomes shorter than in the monomer by 0.041 Å, but remains non-covalent tetrel (NCT). The optimization in a polar medium shortens both bonds to 1.894 and 2.125 Å, respectively, and the N–Si bond is a covalent tetrel (CT). In the second most stable complex (Δ*E* = 1.64 kcal/mol), the water molecule participates in the formation of two H-bonds NH∙∙∙OH 1.999 and OH∙∙∙O=S 2.021 Å, and the N∙∙∙Si bond is lengthened by 0.015 Å compared to the **4d** molecule. Upon calculations in a polar medium, both bonds are shortened compared to the calculation in a gas: the intermolecular H-bond by 0.1 Å, and the intramolecular N–Si by 0.15 Å. Shortening of the non-covalent N∙∙∙Si bond to 2.281 Å in gas and the CTB in a polar medium to 2.135 Å while preserving the nature of each occurs upon the formation of the intermolecular bond OH∙∙∙O=S 2.038 (gas) and 1.952 Å (PCM) by a water molecule. During the interaction of a water molecule with compound **5a** (Figure 9b), the complex formed by two H-bonds, OH∙∙∙O=S 2.004 Å and a weaker CH∙∙∙OH 2.531 Å, which turns out to be more energetically stable in the gas calculation, which can increase the energy of its formation. For calculation in a polar medium, only the OH∙∙∙O=S bond is formed, shortening to 1.962 Å. The bond between the nitrogen and silicon atoms of this complex is shortened to 2.352 Å compared to the monomeric molecule **5a** when calculated in gas, and remains almost unchanged (2.191 Å, PCM), its nature being intact in both cases. The formation of the OH∙∙∙O-Si bond by water at 1.984 (gas) and 1.875 Å (PCM) reduces the N–Si bond to 2.344 (gas) and 2.178 Å (PCM), also without changing its nature.

The addition of the electron-donating DMSO molecule to **4d** (Table 2, Figure 9) involves the formation of H-bond NH∙∙∙O=S 1.792 (gas) and a stronger bond 1.783 Å (PCM). In addition, the oxygen atom of the SO_2_ group in silatrane forms an H-bond with the hydrogen atom of DMSO CH∙∙∙O=S 2.399 (gas) and a weaker bond 2.554 Å (PCM), closing the six-membered cycle. The bonds between the nitrogen and silicon atoms in silatrane are lengthened by 0.05 and 0.01 Å upon calculation in gas and polar medium, respectively, their nature remaining intact compared to the original monomer molecule.

### 2.4. Geometry of ***4d***-di1, ***4d***-di2 and ***5a***-di Dimers and Their Complexes with H_2_O and DMSO Molecules Calculated in Gas and Polar Environment

**Figure 10:**
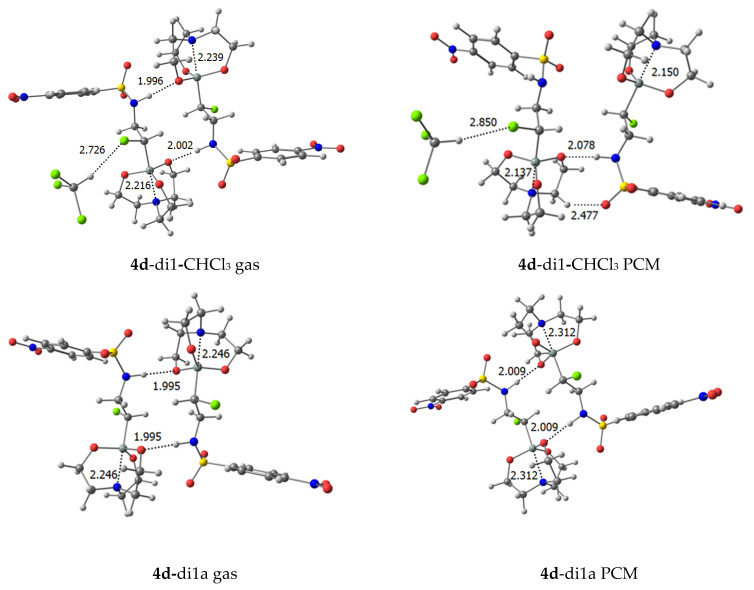

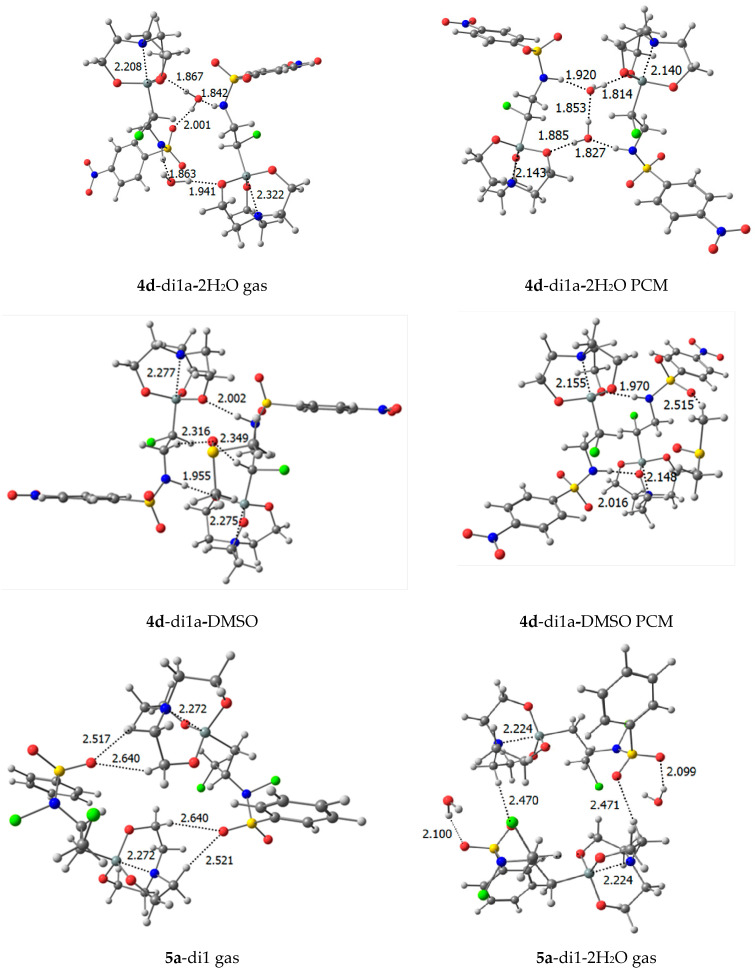
Calculated gas and polar medium (PCM) dimers **4d**-di1, **4d**-di1a and **5a**-di1, and their complexes with two molecules, H_2_O and DMSO. The length of bonds is given in angstroms (Å).

The change in the geometric parameters of cyclic dimers **4d**-di1, **4d**-di2 as well as in dimer **5a**-di depending on the phase state and medium was also analyzed by the comparison of X-ray diffraction data and the results of quantum chemical calculations in gas and a polar medium (Figure 10).

The calculation (in gas) of cyclic dimer **4d**-di1 containing the chloroform molecule shows that the length of both hydrogen bonds NH∙∙∙O-Si decreases compared to the X-ray data by 0.4–0.5 Å, and that of the CH∙∙∙O=S bonds reduces by 0.1 Å. The N–Si bonds are lengthened to 2.239 and 2.216 Å and become NCT N∙∙∙Si ones. The calculation in a polar medium indicates that the distance of one of the H-bonds NH∙∙∙O-Si is greater than the sum of the van der Waals radii of the atoms that form these bonds. As a consequence, this also happens with the neighboring short contact. The second NH∙∙∙O-Si bond is lengthened by 0.08 Å compared to the gas, while the CH∙∙∙O=S bond is shortened by 0.04 Å. Such differences in the lengths of similar intermolecular H-bonds and intramolecular N–Si bonds can only be explained by the presence of a chloroform molecule. The short CH∙∙∙O-Si contact of 2.524 Å formed by chloroform in the crystal compound is elongated when calculated in the gas compared to the data of X-ray diffraction analysis, and its length becomes greater than the sum of the van der Waals radii. However, chloroform forms a non-covalent intermolecular hydrogen bond CH∙∙∙Cl, the length of which (2.726 Å) is slightly less than the sum of the van der Waals radii of the hydrogen and chlorine atoms (2.95 Å). The calculation in a polar medium shows that this bond is elongated to 2.850 Å. On the contrary, the N–Si bonds in this medium are significantly strengthened: they are shortened to 2.150 and 2.137 Å and become CT. In dimer **4d**-di2 containing no chloroform molecules (calculation in gas), the NH∙∙∙O–Si bonds remain almost unchanged as compared to **4d**-di1, and the short CH∙∙∙O=S contacts decrease by 0.1 Å. The N∙∙∙Si bonds are lengthened by 0.01 and 0.03 Å and remain non-covalent tetrel. The calculation of **4d**-di2 in a polar medium indicates that these bonds, as in **4d**-di1, are shortened to 2.141 Å. This value is close to the bond length in the crystal compound and the bond is CT. The lengths of the intermolecular hydrogen bonds NH∙∙∙O-Si (1.991 Å) become close to those of both dimers calculated in the gas, but shorter than in their crystal state. Thus, a short contact of chloroform with the cyclic dimer **4d**-di1 had a slight effect on its intermolecular H-bonds and intramolecular N–Si bonds when calculated both in the gas and in the polar medium. In cyclic dimer **5a**-di1 (calculations in gas), the intermolecular H-bonds CH∙∙∙O=S 2.517 and 2.521 Å become shorter than the short contacts 2.670 Å of the crystal compound, and additional similar bonds 2.640 Å are formed, which are also shorter than the sum of the van der Waals radii of H and O atoms (2.72 Å). The N∙∙∙Si bonds, like in the dimers of compound **4d**, remain NCT, but they are elongated to 2.272 Å. The short contacts of the halogen bond CCl∙∙∙O=S in dimer **5a**-di2 (calculations in gas) become much larger than the sum of van der Waals radii of chlorine and oxygen atoms (3.15 Å) and the dimer breaks. Both dimers of compound **5a** break upon calculation in a polar medium.

The calculations in gas and polar medium show that two water molecules are added to dimer **4d**-di1a, which does not contain a chloroform molecule, and are incorporated into its structure to form the hydrogen bonds NH∙∙∙OH and OH∙∙∙O-Si (Figure 10). The hydrogen atom of water, which is not involved in the formation of in these bonds, remains free (calculations in gas), and their lengths (1.84–1.94 Å) are not strictly symmetrical, unlike the lengths in the original dimer. One of the non-covalent N∙∙∙Si bonds (2.246 Å) in the dimer is shortened to 2.208 Å in accordance with both neighboring shorter H-bonds, and the second is lengthened to 2.322 Å, when both neighboring H-bonds are longer. It should be noted that both N∙∙∙Si bonds remain NCT. When calculating this complex in a polar medium, the intermolecular H-bonds with the water molecule are the same as those calculated in gas. The main difference is that between the water molecules involved in these bonds, the OH∙∙∙OH bond is formed, the length of which (1.853 Å) is in the same range as the others. The N–Si bonds of similar length (2.140 and 2.143 Å) do not change compared to those of the dimer in the polar medium and remain covalent. This may be due to the fact that in the H-complex, the NH∙∙∙OH and OH∙∙∙O-Si bonds close to one silatranyl ring are elongated and shortened, respectively, while the reversed effect in a pair of bonds in the second ring is mutually compensated.

The calculation of the DMSO complex with **4d**-di1 dimer in gas demonstrates that the DMSO molecule, unlike H_2_O, is not added to the NH group, competing with the intermolecular H-bond. At the same time, the oxygen atom of DMSO is involved in the formation of the intermolecular bifurcation bond N-CH∙∙∙O∙∙∙HC-Cl with distances N-CH∙∙∙O=S 2.316 and Cl-CH∙∙∙O=S 2.349 Å.

### 2.5. Wavefunction Analysis

The additional criterion of the nature of the intramolecular bonds formed by the silicon atom with the nitrogen atom and intermolecular hydrogen bonds was obtained from QTAIM analysis [43]. Table 2 and Table 3 summarizes the topological features of the bond critical points (BCPs): the electron density *ρ*(r_c_), the Laplacian of electron density ∇^2^*ρ*(r_c_) and the energies of bonds (*E*), which were calculated as *E* = 1/2V_c_; V_c_ = 1/4∇^2^ρ(r_c_) − 2*G*_c_, where *G*_c_ is the local kinetic electron energy density [44]. The analysis was performed for compounds **4d** and **5a**, which, judging from the geometric parameters of these bonds in gas and in DMSO (PCM), can form both covalent and non-covalent tetrel bonds. The changes in the TB energies and the energies of intermolecular H-bonds formed by H_2_O and DMSO molecules were compared.

As follows from Table 2, the values of *ρ*(r_c_) and ∇^2^*ρ*(r_c_) at the BCPs between the silicon and nitrogen in silatranes **4d**, **5a** and their H-complexes with H_2_O and DMSO in gas are ≤0.05 au corresponding to NCTB N∙∙∙Si. This corresponds to their lengths of 2.289 and 2.390 Å, respectively. Their energy varies within 12–19 kcal/mol, increasing with shortening of the bond. The situation is different in polar medium (PCM). The N–Si bonds are shortened and become CTBs for *ρ*(r_c_) and ∇^2^*ρ*(r_c_) ≥ 0.06 au with an energy of 23–28 kcal/mol. The energy of intermolecular H-bonds with H_2_O varies within 5–8 kcal/mol and is also inversely proportional to the length. At the H-complexes with DMSO, H-bonds shorten to 1.78–1.79 Å and their energy increases to 10 kcal/mol. The values of *ρ*(r_c_) and ∇^2^*ρ*(r_c_) ≤ 0.05 au at the BCPs between the silicon and nitrogen atoms of the dimers **4d**-di1a and **5a**-di1 (Table 3) in gas correspond to non-covalent N∙∙∙Si TBs like their monomeric molecules. In polar medium, the values of *ρ*(r_c_) and ∇^2^*ρ*(r_c_) ≥ 0.06 au correspond to a covalent TB.

Two molecules of water were added to a molecule of dimer **4d**-di1a. These two molecules cleave to the intermolecular NH∙∙∙OSi bonds of the dimer. In this case, each water molecule forms OH∙∙∙OSi (1.867 Å and 1.941 Å) NH∙∙∙OH (1.842 Å) and NH∙∙∙OH (1.863 Å) bonds. The difference in their lengths is due to the fact that one of the water molecules forms an OH∙∙∙O=S bond (2.001) by its second hydrogen atom (Figure 10). The N∙∙∙Si distance in the molecule, in which the above bond is formed, is shortened to 2.208 Å, while the second N∙∙∙Si bond is lengthened to 2.322. In the polar medium, unlike gas, the intermolecular H-bonds OH∙∙∙OSi are shortened to 1.814 and 1.885 Å, and the NH∙∙∙OH bonds are shortened and lengthened to 1.827 and 1.919 Å, respectively. In dimer **4d**-di1a-2H_2_O, the bifurcation OH∙∙∙O=S bond is not formed (the polar medium). This may be due to the formation of a bifurcation bond of the oxygen atom participating in the OH∙∙∙OH (1.853 Å) bond by a proton of water (Figure 10). This can explain the difference in the lengths of the N–Si bonds (2.140 Å and 2.143 Å), which have been shortened in the polar medium. According to the electron distribution in dimer **4d**-di1a with two H_2_O molecules, the N–Si bonds in gas and in DMSO are close to covalent TBs. Despite the length of the N–Si bond (2.2. 2.3 Å), which could be attributed to NCBT, the values *ρ*(r_c_) and ∇^2^*ρ*(r_c_) ≥ 0.06 au with the energy of 26 kcal/mol correspond to covalent N–Si TBs.

## 3. Methods

Calculations, including Quantum Theory of Atom in Molecule (QTAIM) [43] analysis, were performed with the Gaussian09 program package [45]. Geometry optimization of compounds was carried out through the density functional theory method DFT B3PW91 [35,46] and basis set DGDZVP [47]. This method perfectly reproduces the known experimental gas-phase geometries of silatranes. The belonging of stationary points on the potential energy surface to minima was proved by positive eigenvalues of the corresponding Hessian matrices. The Integral Equation Formalism Polarizable Continuum Model (IEF-PCM) with DMSO as the solvent was employed to take into account the solvent polarity. The QTAIM analysis was performed using the AIM2000 program (version 2.0) [48]. Molecular electrostatic potentials were calculated at the MP2/aug-cc-pVDZ level [28,42,49], and analyzed with the Multiwfn 3.3.5 program [50] on the 0.001 au electron density isosurface.

## 4. Conclusions

According to the X-ray diffraction analysis of sulfonamide derivatives of silatrane N-[2-chloro-2-(silatranyl)ethyl]-4-nitro-benzenesulfonamide **4d** and N-chloro-N-[2-chloro-1-(silatran-1-yl-methyl)ethyl] benzenesulfonamide **5a**, it was established that their supramolecular structure is formed by cyclic dimers of two types. An independent crystal cell of silatrane **4d** contains two molecules that are linked by intermolecular hydrogen bonds NH∙∙∙O-Si and CH∙∙∙O=S short contacts. The supramolecular structure of silatrane **5a** is formed via the intermolecular short contacts N–CH∙∙∙O=S and C–Cl∙∙∙O=S. The cyclic dimers **4d** and **5a** are formed via short contacts to infinite chains. These chains are arranged in layers that are not bonded to each other. The length of intramolecular N–Si bonds of those compounds, 2.08–2.14 Å, corresponds to their covalent tetrel nature. They remain covalent tetrel in calculation of compounds **4d** and **5a** and their dimers in the polar medium, but are significantly lengthened, becoming noncovalent tetrel N∙∙∙Si upon calculation in gas. An increase in the polarity of the medium does not change the distribution of MESPs. According to the QTAIM analysis of silatranes **4d**, **5a**, their cyclic dimers, which are the basis of the supramolecular structure, the energies of N∙∙∙Si and N–Si bonds vary within 12–19 kcal/mol in gas and 23–28 kcal/mol in solution, respectively. The formation of donor–acceptor complexes of these compounds with molecules of water and DMSO affects the strength of their N∙∙∙Si and N–Si bonds, but does not change their nature. The obtained results allow us to assume their possible participation in chemical processes in the low-polarity environment of the silatranyl cycle of compounds **4d** and **5a** upon changes in their biological activity.

## Figures and Tables

**Table 1:** Most negative (V_min_, kcal/mol) and most positive (V_max_, kcal/mol) molecular electrostatic potentials (MESPs) on MESP maps and lengths (Å) of intramolecular N–Si bonds of compounds **4d**, **5a** and **6**–**9**, calculated in gas phase and solvent (PCM, italic) and from X-ray analysis (**bold**).

Entry	O=S	O-(Si)	H-N	Si	CH_2_O-Si	Cl-CH_2_	*l* (N–Si)
**4d**	−42.06	−32.62	30.73	6.31	33.95	−5.52	2.289
	−40.71	−27.22					
*PCM*	*−44.75*	*−35.88*	*30.01*	*3.27*	*39.25*		
	*−43.34*	*−30.88*			*36.36*	*−9.35*	*2.143*
**X-ray**							**2.036**
**5a**	−47.87 −45.96	−21.26	*−*	*−*	31.24 31.49	−12.77	2.390
*PCM*	*−51.55* *−50.09*	*−43.39* *−28.66*	*−*	*−*	*36.57* *35.46*	*−15.86*	*2.193*
**X-ray**							**2.136**
**6**	−39.24 −37.60	−33.08 −27.33	32.76	7.00	32.83 28.42	−6.26	2.297
*PCM*	*−41.84* *−40.14*	*−36.33* *−31.65*	*32.41*	*5.47*	*38.91* *36.20*	*−10.15*	*2.138*
**7**	−49.60 −48.18	−35.22 −27.99 −25.18	24.44	3.52	31.57 26.37	−7.91	2.310
*PCM*	*−52.66* *−51.39*	*−38.85* *−32.72* *−27.85*	*23.43*	*−*	*37.35* *35.74* *35.29*	*−12.41*	*2.146*
**8**	−31.49 −29.58		46.19				*−*
**9**	−32.83 −32.05		54.62				*−*

**Table 2:** Calculated gas and solvent (PCM) bond lengths (*l*, Å, *ρ*(**r**_c_), ∇^2^*ρ*(**r**_c_), au) at the BCPs, and bond energies (*E*, kcal/mol) in molecule **4d** and her H-complexes with H_2_O (**4d**-I-**4d**-III) and DMSO (IV) molecules and in molecule **5a** and her H-complexes with H_2_O (**5a**-V, **5a**-VI).

Entry	Bond	*l*	*ρ*(r_c_)	∇^2^*ρ*(r_c_)	*E*	Bond	*l*	*ρ*(r_c_)	∇^2^*ρ*(r_c_)	*E*
	Gas					PCM				
**4d**	N∙∙∙Si	2.289	0.050	0.037	16.6	N–Si	2.143	0.058	0.075	23.0
**4d**-I H_2_O	N∙∙∙Si OH∙∙∙O-Si	2.248 2.050	0.053 0.020	0.049 0.080	19.1 5.1	N–Si OH∙∙∙OSi	2.125 1.894	0.064 0.028	0.105 0.107	27.7 7.6
**4d**-II H_2_O	N∙∙∙Si NH∙∙∙OH OH∙∙∙O=S	2.304 1.999 2.021	0.049 0.024 0.021	0.034 0.091 0.084	15.9 5.8 5.3	N–Si NH∙∙∙OH OH∙∙∙O=S	2.152 1.897 –	0.061 0.029 –	0.091 0.091 –	25.5 7.7 –
**4d**-III DMSO	N∙∙∙Si OH∙∙∙O=S	2.281 2.038	0.050 0.019	0.039 0.076	17.1 4.6	N–Si OH∙∙∙O=S	2.139 1.952	0.063 0.023	0.098 0.093	26.6 6.0
**4d**-IV DMSO	N∙∙∙Si NH∙∙∙O=S	2.341 1.792	0.045 0.037	0.029 0.129	14.0 10.3	N–Si NH∙∙∙O=S	2.153 1.783	0.061 0.037	0.091 0.131	26.3 10.6
**5a**	N∙∙∙Si	2.390	0.042	0.026	12.0	N–Si	2.193	0.057	0.072	22.4
**5a**-V H_2_O	N∙∙∙Si OH∙∙∙O-Si	2.344 1.984	0.046 0.023	0.028 0.088	13.9 5.8	N–Si OH∙∙∙OSi	2.178 1.875	0.058 0.030	0.080 0.088	23.5 8.0
**5a**-VI H_2_O	N∙∙∙Si OH∙∙∙O=S	2.352 2.004	0.045 0.020	0.028 0.082	13.5 4.9	N–Si OH∙∙∙O=S	2.191 1.962	0.057 0.022	0.073 0.091	22.6 5.8

**Table 3:** Calculated gas and solvent (PCM) bond lengths (*l*, Å), *ρ*(**r**_c_), ∇^2^*ρ*(**r**_c_, au) at the BCPs, and bond energies (*E*, kcal/mol) in H-complexes of dimers of **4d**-di1a, **5a**-di1 and **4d**-di1a with 2H_2_O.

Entry	Bond	*l*	*ρ*(r_c_)	∇^2^*ρ*(r_c_)	*E*	Bond	*l*	*ρ*(r_c_)	∇^2^*ρ*(r_c_)	*E*
	Gas					PCM				
**4d**-di1a	N∙∙∙Si N∙∙∙Si NH∙∙∙O-Si NH∙∙∙O-Si	2.246 2.246 1.995 1.995	0.053 0.053 0.023 0.023	0.048 0.048 0.088 0.088	19.3 19.3 5.7 5.7	N–Si N–Si NH∙∙∙O-Si NH∙∙∙O-Si	2.141 2.141 1.991 1.991	0.063 0.063 0.024 0.024	0.095 0.095 0.089 0.089	26.5 26.5 5.8 5.8
**4d**-di1a 2H_2_O	N∙∙∙Si N∙∙∙Si NH∙∙∙OH NH∙∙∙OH OH∙∙∙O-Si OH∙∙∙O-Si	2.208 2.322 1.842 1.863 1.867 1.941	0.063 0.063 0.031 0.038 0.035 0.029	0.085 0.084 0.116 0.134 0.122 0.106	26.7 26.4 8.3 11.0 9.7 7.7	N–Si N–Si NH∙∙∙OH NH∙∙∙OH OH∙∙∙O-Si OH∙∙∙O-Si	2.140 2.143 1.827 1.920 1.814 1.885	0.063 0.062 0.035 0.028 0.034 0.029	0.096 0.094 0.124 0.104 0.120 0.107	25.7 26.8 9.5 7.2 9.4 7.8
**5a**-di1	N∙∙∙Si CH∙∙∙ O=S CH∙∙∙O=S N∙∙∙Si CH∙∙∙O=S CH∙∙∙O=S	2.272 2.517 2.640 2.272 2.521 2.640	0.051 0.007 0.004 0.051 0.007 0.004	0.041 0.029 0.015 0.041 0.029 0.015	17.7 1.4 0.7 17.7 1.4 0.7					

## Data Availability

Data are contained within the article.
